# Age and Diet Affect Gene Expression Profile in Canine Skeletal Muscle

**DOI:** 10.1371/journal.pone.0004481

**Published:** 2009-02-16

**Authors:** Ingmar S. Middelbos, Brittany M. Vester, Lisa K. Karr-Lilienthal, Lawrence B. Schook, Kelly S. Swanson

**Affiliations:** 1 Department of Animal Sciences, University of Illinois, Urbana, Illinois, United States of America; 2 Division of Nutritional Sciences, University of Illinois, Urbana, Illinois, United States of America; 3 Department of Veterinary Clinical Medicine, University of Illinois, Urbana, Illinois, United States of America; Brunel University, United Kingdom

## Abstract

We evaluated gene transcription in canine skeletal muscle (biceps femoris) using microarray analysis to identify effects of age and diet on gene expression. Twelve female beagles were used (six 1-year olds and six 12-year olds) and they were fed one of two experimental diets for 12 months. One diet contained primarily plant-based protein sources (PPB), whereas the second diet contained primarily animal-based protein sources (APB). Affymetrix GeneChip Canine Genome Arrays were used to hybridize extracted RNA. Age had the greatest effect on gene transcription (262 differentially expressed genes), whereas the effect of diet was relatively small (22 differentially expressed genes). Effects of age (regardless of diet) were most notable on genes related to metabolism, cell cycle and cell development, and transcription function. All these genes were predominantly down-regulated in geriatric dogs. Age-affected genes that were differentially expressed on only one of two diets were primarily noted in the PPB diet group (144/165 genes). Again, genes related to cell cycle (22/35) and metabolism (15/19) had predominantly decreased transcription in geriatric dogs, but 6/8 genes related to muscle development had increased expression. Effects of diet on muscle gene expression were mostly noted in geriatric dogs, but no consistent patterns in transcription were observed. The insight these data provide into gene expression profiles of canine skeletal muscle as affected by age, could serve as a foundation for future research pertaining to age-related muscle diseases.

## Introduction

Aging mammals display a decline in a multitude of physical and physiological functions. In addition to impaired cognitive function [1], [2] with age, muscle function and strength may also decline [3]. The decline in muscle function in aging dogs is attributed to oxidative damage to lipids, proteins, and DNA that accumulates over time [4]. In metabolically active muscle tissue, mitochondrial DNA damage leads to dysfunction [5] and may lower oxygen uptake capacity thus decreasing muscle function [6]. Decreased expression of genes related to the electron transport chain, energy metabolism, and mitochondrial protein synthesis have been reported in aged human skeletal muscle [7], [8].

Typical dietary effects on gene expression are noted with caloric restriction, which not only slows the aging process, but also mediates the transcription of metabolic and biosynthetic genes [9]. Additionally, in a calorically restricted state, mitochondria have been reported to decrease oxygen consumption, generate fewer reactive oxygen species, and maintain critical ATP production [10]. Other dietary manipulations, including differences in protein source, have also been shown to affect hepatic and skeletal muscle gene expression in rats [11].

Muscle gene expression in dogs has been evaluated for some select genes under pathogenic [12] and varying dietary conditions [13] but no large-scale profiling data are available. Therefore, the aim of this study was to investigate the effects of age and dietary composition on gene expression in skeletal muscle of dogs. This experiment was part of a larger study investigating the effects of age and dietary composition on various physiologic and genomic outcomes. We previously demonstrated that diet and age affected whole body metabolism [14], intestinal morphology and fermentative end-products [15], and cerebral cortex gene expression [16].

## Results and Discussion

Diets fed in this study were previously reported to affect nutrient digestibility [14], gut morphology [15], and gene transcription of cerebral cortex tissue [16]. Age was reported to have the greatest effect on cerebral cortex gene expression, whereas the effects of diet were relatively small. Geriatric dogs had increased expression of genes related to inflammation and stress response, as well as calcium homeostasis, whereas gene expression related to neurotransmission was decreased [16].

In canine skeletal muscle, age had the strongest effect on mRNA abundance, whereas the effect of diet was less pronounced. A total of 390 probe sets were significantly changed with age in either pairwise comparison (old vs. young fed APB; old vs. young fed PPB), whereas only 30 probe sets were significantly changed due to diet in either pairwise comparison (APB vs. PPB in old dogs; APB vs. PPB in young dogs). After eliminating probe sets that represented unannotated genes and correcting for multiple probe sets that represented the same gene, 262 genes were differentially expressed due to age, whereas 22 genes were differentially expressed due to diet. All microarray data have been deposited in the Gene Expression Omnibus repository at the National Center for Biotechnology Information archives (http://www.ncbi.nlm.nih.gov/geo) under accession #GSE12502.

**Figure 1:**
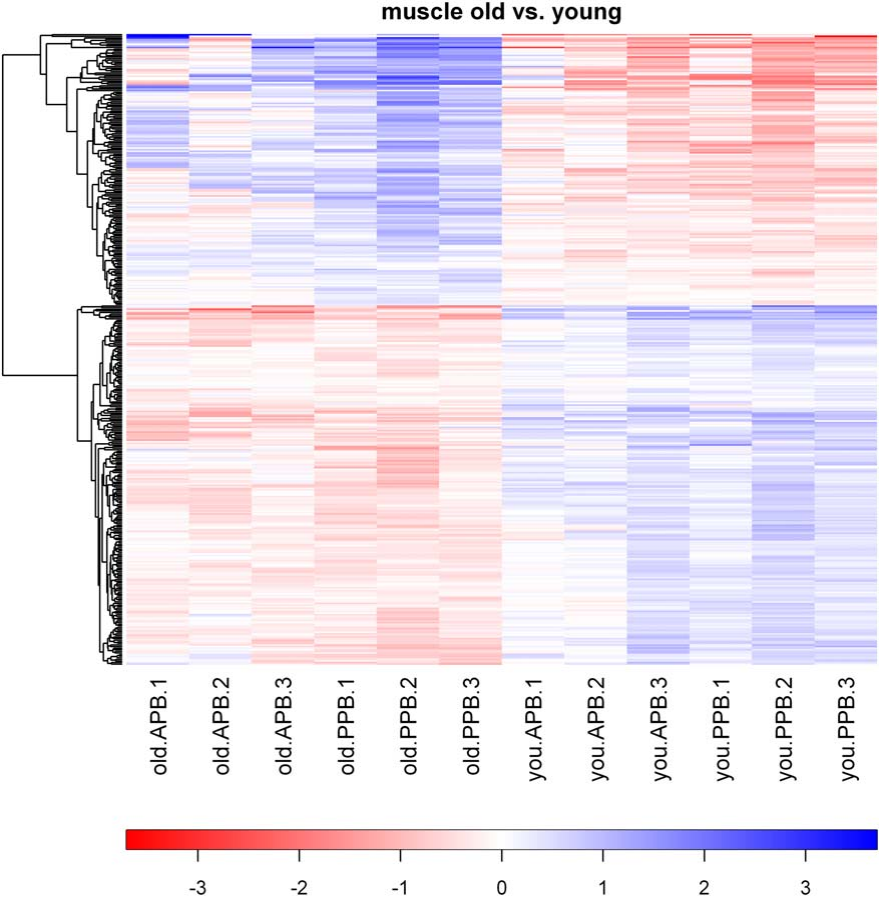
Heat map of pairwise comparisons of skeletal muscle gene expression in geriatric versus young adult dogs. Values are the GCRMA-processed probe set value (log2 scale) minus the mean value for that probe set across all arrays. Hierarchical cluster analysis was used to create the dendrogram.

The heat map in Figure 1 clearly demonstrates the strong and consistent effect of age, but also highlights some inconsistencies, particularly in geriatric dogs fed the APB diet. Although age was the primary factor by which dogs were clustered, dogs also clustered according to diet within age groups. Of the 262 genes that were affected by age, only 26 were differentially expressed in both diet groups (Tables 1, 2, and 3). Of the total 22 differentially expressed genes due to diet, none were differentially expressed in both age groups (Table 4). Analysis of several genes using qRT-PCR confirmed our observations on the array, with MT2A, TTN, and ATP2B all having increased mRNA abundance according to both the array and qRT-PCR (data not shown).

**Table 1:** Differentially expressed cell function- and immune response-associated genes in skeletal muscle tissue of geriatric vs. young adult dogs fed diets formulated with animal-based (APB) or plant-based (PPB) protein sources.

Functional classification	Gene name	Gene symbol	Fold change	
			APB	PPB
**Cell cycle and apoptosis**				
Apoptosis	TIMP metallopeptidase inhibitor 3	TIMP3	1.36	1.90*
Apoptosis	Diablo homolog (drosophila)	DIABLO	−1.25	−2.18*
Cell adhesion	Endoglin (osler-rendu-weber syndrome 1)	ENG	−2.85*	−3.61*
Cell aging	LIM and senescent cell antigen-like domains 1	LIMS1	1.31	3.13*
Cell cycle	Stromal interaction molecule 1	STIM1	−1.60*	−1.95*
Cell cycle	Transforming growth factor, beta 1	TGFB1	−1.54	−2.12*
Cell cycle (inhibition)	Retinoblastoma-like 2 (p130)	RBL2	1.61	3.45*
Cell proliferation	RAS homolog gene family, member g (rho g)	RHOG	−1.04	−1.65*
Cell survival	Hcls1 associated protein x-1	HAX1	−1.56*	−1.80*
Differentiation	Endothelial pas domain protein 1	EPAS1	−1.69*	−2.10*
Meiotic recombination	Kelch domain containing 3	KLHDC3	−1.57*	−1.77*
Proliferation	Calpain, small subunit 1	CAPNS1	−1.36*	−1.41*
**Cellular organization and development**				
Actin cytoskeleton organization and development	Phosphodiesterase 4 d interacting protein (myomegalin)	PDE4DIP	1.30	1.90*
Cytokinesis	Myosin-10 (cellular myosin heavy chain, type b)	MYH10	3.19	6.22*
Cytokinesis	Septin 11	SEPT11	2.52*	3.79*
Cytoskeletal organization	Actin binding lim protein 1	ABLIM1	2.23	3.17*
Development	MYBPC1 myosin binding protein C, slow type	MYBPC1	1.82	4.14*
Development	Nebulin	NEB	1.86	3.16*
Development	Titin	TTN	1.96	8.95*
Development	Myocyte enhancer factor 2C	MEF2C	1.38	4.90*
Development	Integrin beta 1 binding protein (melusin) 2	ITGB1BP2	−2.02	−2.28*
Development	Actin, gamma 2, smooth muscle, enteric	ACTG2	−2.72	−6.73*
Structure	DMN desmuslin	DMN	2.13	8.41*
**Immune and stress response**				
Anti tumor	Dermatan sulfate epimerase	DSE	4.81*	4.49*
Humoral immune response	Adenosine deaminase	ADA	−2.21*	−2.58*
Immune response	Gamma-interferon inducible lysosomal thiol reductase precursor	IFI30/GILT	−4.70*	−5.30*
Oxidative stress response	Peroxiredoxin 5	PRDX5	−1.90*	−1.90*

* Denotes statistically significant differences.

**Table 2:** Differentially expressed metabolism-associated genes in skeletal muscle tissue of geriatric vs. young adult dogs fed diets formulated with animal-based (APB) or plant-based (PPB) protein sources.

Functional classification	Gene name	Gene symbol	Fold change	
			APB	PPB
**Metabolism**				
Amino acid metabolism	Cysteine conjugate-beta lyase; cytoplasmic (glutamine transaminase K, kyneurenine aminotransferase)	CCBL1	−2.48*	−2.08*
Carbohydrate metabolism	ER degradation enhancer, mannosidase alpha-like 2	EDEM2	−1.36*	−1.66*
Electron transport	NADH dehydrogenase (ubiquinone) 1 beta subcomplex, 9, 22 kDa	DNDUFB9	−1.29	−1.37*
Electron transport	Isovaleryl Coenzyme A dehydrogenase	IVD	−1.50	−1.69*
Electron transport	NADH dehydrogenase (ubiquinone) Fe-S protein 6, 13 kDa (NADH-coenzyme Q reductase)	NDUFS6	−1.22	−1.42*
Electron transport chain	NADH dehydrogenase 1 beta subcomplex, 11, 17.3 kda	NDUFB11	−1.61*	−1.71*
Electron transport chain	Ring finger 144b	RNF144B	−2.79*	−2.56*
Electron transport chain	Glutaryl-Coenzyme A dehydrogenase	GCDH	−2.01*	−1.50
Energy metabolism	NADH dehydrogenase (ubiquinone) 1 alpha subcomplex, 10, 42 kDa	NDUFA10	−1.66*	−1.46
Lipid metabolism	Protein kinase, AMP-activated, gamma 1 non-catalytic subunit	PRKAG1	−1.41	−2.10*
Glutathione conjugation	Glutathione S-transferase P (GST 7-7) (Chain 7) (GST class-pi)	GSTP1	−1.22	−1.77*
Glycolysis	Glyceraldehyde-3-phosphate dehydrogenase	GAPDH	−1.13	−2.13*
Glycolysis	Aldolase A, fructose-bisphosphate	ALDOA	1.53	2.54*
Heme biosynthesis	Heme a∶farnesyltransferase	COX10	−1.57*	−1.54*
Lipid metabolism	Hydroxysteroid (17-beta) dehydrogenase 10	HSD17B10	−1.76*	−1.83*
Protein AA dephosphorylation	Protein phosphatase 2, regulatory subunit B″, alpha	PPP2R3A	1.30	3.67*
Protein AA phosphorylation	Janus kinase 1 (a protein tyrosine kinase)	JAK1	1.66	3.64*
TCA cycle	Isocitrate dehydrogenase 3 (NAD+) beta	IDH3B	−2.19*	−2.23*
TCA cycle	Succinate dehydrogenase complex, subunit C, integral membrane protein, 15 kDa	SDHC	−1.43*	−1.34

* Denotes statistically significant differences.

**Table 3:** Differential expression of transcription-, signaling-, and transport-associated genes in skeletal muscle tissue of geriatric vs. young adult dogs fed diets formulated with animal-based (APB) or plant-based (PPB) protein sources.

Functional classification	Gene name	Gene symbol	Fold change	
			APB	PPB
**Transcription - translation**				
DNA structure	Polymerase (DNA-directed), epsilon 4 (p12 subunit)	POLE4	−1.20*	−1.32*
mRNA processing	Small nuclear ribonucleoprotein polypeptide N	SNRPN	−1.54*	−1.73*
Protein biosynthesis	Deoxyhypusine synthase	DHPS	−1.45	−2.03*
Protein biosynthesis	Mitochondrial ribosomal protein L14	MRPL14	−1.37	−1.65*
Protein biosynthesis	Mitochondrial ribosomal protein S18A	MRPS18A	−1.34	−1.72*
Protein biosynthesis	Ribosomal protein S16	RPS16	−1.15	−2.00*
Protein biosynthesis	Ribosomal protein L19	RPL19	−1.03	−1.67*
Protein biosynthesis	Mitochondrial ribosomal protein S12	MRPS12	−1.42	−1.75*
Ribosome assembly	Nucleolar protein family a, member 2	NOLA2	−1.36*	−1.89*
Transcription regulation	Zinc finger protein 32	ZNF32	−1.63*	−1.75*
**Signaling mechanisms**				
Cellular trafficking	Methylmalonic aciduria cblc type, with homocystinuria	MMACHC	−2.21*	−2.15*
Intracellular signaling cascade	A kinase (prka) anchor protein 13	AKAP13	1.96*	1.86*
Neurotransmission	Rab3a, member ras oncogene family	RAB3A	−2.27*	−2.22*
**Transport**				
Protein transport	Sec61 alpha 2 subunit (s. Cerevisiae)	SEC61A2	−1.54*	−1.45*
Taurine transport	Solute carrier family 6 (neurotransmitter transporter), member 6	SLC6A6	1.87*	2.40*
Transport	Solute carrier family 25, member 33	SLC25A33	−3.14*	−3.42*

* Denotes statistically significant differences.

**Table 4:** Effect of an animal protein-based vs. a plant protein-based diet on muscle gene expression in young adult or geriatric dogs.

Functional classification	Gene name	Gene symbol	Fold change	
			Young	Geriatric
**Cellular organization and development**				
Muscle function and development	Titin	TTN	1.16	−4.77*
Striated muscle development	Myosin binding protein C, slow type	MYBPC1	−1.02	−1.46*
**Metabolism**				
Diabetes and obesity	Fat mass and obesity associated	FTO	−1.07	−1.39*
Electron transport	Monoamine oxidase B	MAOB	−2.61*	1.05
Glycolysis	Glyceraldehyde-3-phosphate dehydrogenase	GAPDH	1.06	2.01*
Leucine catabolism	Methylcrotonyl-Coenzyme A carboxylase 2 (beta)	MCCC2	1.91*	1.11
**Transcription - translation**				
Transcription regulation	Zinc finger, MYND-type containing 8	ZMYND8	−1.07	−1.69*
Transcription regulation	SNF8, ESCRT-II complex subunit	SNF8	−1.34*	−1.02
**Signaling mechanisms**				
Cell surface receptor-linked Signal transduction	Leukemia inhibitory factor receptor alpha	LIFR	1.05	−2.20*
Intracellular signaling cascade	Janus kinase 1 (a protein tyrosine kinase)	JAK1	−1.12	−2.17*
Signal transduction	A kinase (PRKA) anchor protein 8	AKAP8	−1.04	−1.35*
Signal transduction	Guanine nucleotide binding protein (G protein), beta polypeptide 2-like 1	GNB2L1	−1.05	−1.52*
Signal transduction	Fasciculation and elongation protein zeta 2 (zygin II)	FEZ2	−1.52	−1.68*
**Other**				
Apoptosis	Thioredoxin-like 1	TXNL1	1.04	1.29*
Calcium transport	ATPase, Ca++ transporting, plasma membrane 1	ATP2B1	1.01	−1.55*
Cell adhesion	Collagen, type I alpha 2	COL1A2	−1.26	−2.68*
Lipid transport	Glycolipid transfer protein domain containing 1	GLTP	−1.01	2.30*
Mitotic chromosome condensation	Cytoplasmic linker associated protein 2	CLASP2	−1.14	−2.23*
Peptidase	Signal peptide peptidase 3	UNQ1887	−1.59*	1.05
Protein complex assembly	Solute carrier family 9 (Na/H exchanger) member 3 regulator 1	SLC9A3R1	−1.00	1.21*
Unknown	Growth hormone regulated TBC protein 1	GRTP1	1.04	1.37*
Visual perception	Crystallin, zeta	CRYZ	−1.06	−1.38*

* Denotes statistically significant differences.

The expression of genes related to cell cycle and apoptosis in geriatric dogs versus young adults suggests decreased cell turnover and increased apoptotic cells in geriatric individuals. Genes that promote proliferation (e.g., CAPNS1) and differentiation (e.g., EPAS1) had decreased expression in geriatric dogs. On the other hand, genes related to induction of apoptosis, cell aging, and cell cycle inhibition (TIMP3, LIMS1, and RBL2) had increased expression in geriatric dogs. Nevertheless, DIABLO, whose down-regulation (in geriatric dogs fed PPB) suggests decreased apoptosis, contradicts the above described pattern of decreased cell turnover and increased apoptosis. This observation may be due to the method by which each gene induces cell death. TIMP3 stabilizes TNFα receptors on the cell surface, priming the cell for TNF-induced death often seen in cancerous cells [17], whereas DIABLO acts through inhibition of apoptosis inhibitor proteins [18].

Decreased expression of ENG and TGFB1 (in geriatric dogs fed PPB) suggests decreased cell turnover in geriatric dogs. ENG or endoglin is a co-receptor for TGFB1 that controls endothelial cell proliferation and angiogenesis [19]. ENG has been identified in muscle satellite cells or myogenic progenitors (MPs), but in contrast to our findings, Beggs et al. [20] noted increased expression of ENG in aged MPs compared to adult MPs (8-month old mice vs. 23-month old mice).

Interestingly, STIM1, a gene related to cell growth suppression [21], was up-regulated in young adult dogs. STIM1 controls intracellular calcium homeostasis in conjunction with STIM2 [22], [23]. Calcium is an important second messenger, which may affect cell functions, including growth, and, specifically for muscle, contraction. Moreover, calcium is a co-factor for CAPNS1, which controls the stability of calpains (both µ and m) [24]. Therefore, by dependency, calcium (and possibly STIM) may control CAPNS1 and have strong control over cell proliferation and apoptosis. Additionally, the calpain system may play a role in stress or damage response [25].

In general, the up-regulation of genes related to cell cycle and proliferation in young adult dogs is not surprising as they have a higher rate of tissue turnover. For example, HAX1 (decreased in geriatric dogs) was increased in human psoriasis (epidermal hyperplasia) patients, indicating its effect on cell proliferation [26] and suppression of apoptosis [27]. Expression of EPAS1, a hypoxia-induced gene, was also decreased in geriatric dogs. A lack of EPAS1 can have severe metabolic consequences, including multiple organ pathogenesis, dysregulated TCA cycle and fatty acid oxidation, and impaired homeostasis of reactive oxygen species [28]. In the healthy individuals, EPAS1 stimulates angiogenesis [29]. KLHDC3 (Peas in mouse) bears similarity to RAG2 normally found in the testis where it is thought to play a role in meiotic recombination [30], but its function in skeletal muscle is unknown. Collectively, these data suggest that cell cycle, cell proliferation, and cell differentiation were decreased in geriatric dogs versus young adult dogs, whereas apoptosis and cell aging may be increased. The measurement of indicators for apoptosis or cell turnover were not performed herein to confirm the mRNA data, but would be justified in future experiments.

Concurrent with genes related to cell cycle in geriatric dogs, genes related to DNA transcription (e.g., POLE4 and ZNF32) were also down-regulated. Although the function of POLE4 has not been completely elucidated, it is thought to play a role in DNA replication (particularly chromatin folding) [31] in the late S-phase of mitotic cells and is therefore associated with cell proliferation [32]. ZNF32 has been implicated in human colon and adrenocortex cancers where its up-regulation was noted in “signature” gene expression profiles for these malignancies [33], [34]. Implications for ZNF32 expression in skeletal muscle and its relation to age are currently unknown.

SNRPN is involved in mRNA processing and its dysfunction (by faulty imprinting) in humans is related to Prader-Willi and Angelman syndromes [35], which typically impair intelligence, cognitive development, and display low muscle tone (hypotonia). SNRPN expression appears to be ubiquitous in mice, including skeletal muscle [36] but specific information in canine muscle is lacking. Likewise, NOLA2 has not been described in canine skeletal muscle but its involvement in rRNA processing and particularly its role in the telomerase complex have primed NOLA2 as an indicator gene in human lung cancer [37].

A number of genes related to protein biosynthesis were expressed at lower levels in aged dogs compared to young adult dogs, specifically in those fed PPB. Most of these genes encode ribosomal proteins or subunits thereof, but specific functions of these proteins remain to be elucidated. Although they are not functionally categorized as being involved in transcription and translation in Table 3, RBL2 (cell cycle; Table 1) and PPP2R3A (metabolism; Table 2) both play a role in transcription and repress the cell cycle.

The down-regulation of genes related to metabolism in skeletal muscle of geriatric dogs was not surprising, as aged muscle becomes less efficient due to mitochondrial dysfunction [5], [6], [38]. These age-related changes affect respiratory chain function [39] and, consequently, reduce VO_2max_ thereby diminishing muscle function [6] and muscle strength [3]. These age-related changes likely affect genes involved with the respiratory chain (NDUFB11, RNF144B, COX10, IVD, NDUFS6, and GCDH) directly, as well as IDH3B and SDHC, which are part of the citric acid cycle [40], and CCBL1, which is involved in glutamine metabolism [41]. HSD17B10 regulates biological potency of steroid hormones but is also involved in mitochondrial β-oxidation of fatty acids [42]. PRKAG1, which plays a role in fatty acid synthesis, was also decreased geriatric dogs fed PPB. The efficiency of carbohydrate metabolism may be impaired with age due to decreased EDEM2 expression. EDEM2 recognizes misfolded endoplasmic reticulum glycoproteins and targets them for destruction [43], [44], however, a direct effect of age on this gene has not been described to date. In general, our gene expression data suggest reduced metabolic activity with age, which is in agreement with previously reported data in humans [7], [8]. Further studies using calorimetric devices are required to confirm our gene expression data.

It is interesting to note that several genes related to cellular organization and development were up-regulated in geriatric dogs, almost exclusively in those fed PPB. Whereas genes related to cell cycle and proliferation and differentiation suggest a decrease in cell division, these organization and development genes were significantly changed in the opposite direction. This response could be due to age-related muscle atrophy, where fibrous tissue replaces muscle tissue [45]. Moreover, the decreased expression of melusin (ITGB1BP2), which suppresses hypertrophy [46], may indicate an attempt to fill voids left by dead muscle cells. Cell hypertrophy would require an increase in size of the structural proteins (e.g., PDE4DIP, DMN, and ABLIM1) and functional proteins (e.g., MYBPC1, NEB, and TTN), which is in agreement with the observed up-regulation of these genes. Additionally, an increase in expression of genes related to cytokinesis was also noted (SEPT11 and MYH10), further underscoring the possibility of cellular hypertrophy.

As individuals age, oxidative damage to tissues tends to increase. This is especially the case in highly oxidative tissues such as skeletal muscle [4]. Oxidative damage, in turn, leads to the induction of genes that counteract the effects of oxidative stress [9]. Interestingly, a gene such as PRDX5, which has antioxidant activity had lower expression in aged dogs, which is in agreement with observations in aged mouse hearts [47]. ADA was also down-regulated in geriatric dogs, but is typically not highly expressed in skeletal muscle [48]. Although ADA is linked to development of the immune system in humans (deficiency causes severe combined immunodeficiency disorder), specifically in the spleen [48], its function in muscle may be limited to purine metabolism. The up-regulation of DSE (formerly known as SART2) in skeletal muscle of geriatric dogs may be related the oxidative damage in older tissues, as DSE is a tumor antigen that is recognized by cytotoxic T cells and is typically highly expressed in tumor cells [49].

Although no consistent trends were observed, age also affected some genes associated with cell signaling or transport. AKAP13, recently identified as a modulator of Toll-like receptor 2, was up-regulated in geriatric dogs. It has the ability to activate NF-κB and therefore activate the innate immune response [50], which could potentially be a response to age-related increased oxidative damage in muscle cells. Synaptic vesicle function appears to be impaired in geriatric dogs given the down-regulation of RAB3A. This gene plays a role in neurotransmitter release [51], [52] which may be negatively affected as synaptic vesicle density decreases with age [53]. SEC61A2 is an essential part of the endoplasmic reticulum protein translocation complex [54] and its transcription may be regulated in a p53-dependent manner [55]. This latter mechanism may also reciprocally affect SLC6A6 expression, as p53 represses SLC6A6 expression [56]. SLC6A6 is ubiquitously expressed in human tissues [57] and is a sodium dependent taurine transporter that has been implicated in mental retardation in humans [56], but its function in dogs, and specifically skeletal muscle, is not known. The expression of SLC25A33 is fairly ubiquitous in humans, with the highest expression in testis, skeletal muscle [58], and central nervous system [59] but data in the dog are unavailable. As with many members of the large SLC family of carriers, the substrate of SLC25A33 is currently unknown.

Taken together, the age-related changes in gene expression in dogs fed either diet suggest a decreased level of cell development and proliferation, transcription – translation activity, and metabolism. Genes up-regulated with age (mostly in dogs fed PPB) suggest responses to a “troubled” system, initiating hypertrophy of remaining cells in order to avoid muscle tissue from being replaced with non-muscle tissue. It should be noted that a considerably larger number of genes than are listed in Tables 1, 2, and 3 were affected by age. A complete list of genes affected by age is reported in Table S1.

Only four genes were differentially expressed in skeletal muscle of young adult dogs as affected by diet (MCCC2, MAOB, SNF8, and UNQ1887; Table 4). MCCC2 is a carboxylase involved in leucine catabolism [60], while MAOB catalyzes the oxidative deamination of biogenic amines including dopamine, serine, adrenalin and nor-adrenalin [61], [62]. SNF8 (in yeast) has a human homologue known as EAP30 subunit of the ELL complex (a transcription factor) that increases gene expression [63]. The aspartyl protease UNQ1887 (also known as SPPL3) is located in the endoplasmic reticulum and is thought to be involved in the clearance of signal peptides [64].

In geriatric dogs, differential gene expression as affected by diet was more prevalent. Because geriatric dogs are in a “steady-state” as compared to growing dogs, relatively small dietary manipulations may result in larger gene expression changes. More differentially expressed genes were present in geriatric dogs fed the animal-based diet, but consistent patterns did not exist. Because of the reduced caloric density and nutrient digestibility of the PPB diet, dogs fed that treatment had a 55% higher protein intake and consumed 13% more calories compared with dogs fed the APB diet. Conversely, dogs fed the APB diet had a 57% higher fat intake [14]. These differences may have contributed to our results.

Genes associated with muscle contraction were down-regulated in geriatric dogs fed the APB diet. Helman et al. [65] reported that dogs fed diets containing animal protein (chicken) were better able to control calpastatin-related protein degradation than those fed diets containing corn gluten meal. Further research is necessary to determine whether decreased expression of MYBPC1 and TTN in geriatric dogs fed APB as compared to PPB in this experiment are mechanisms by which this occurs. Genes associated with signaling mechanisms were also decreased in geriatric dogs fed the APB diet. Such genes have been reported to function in tissue regeneration (LIFR) [66], cell growth (JAK1 [67] and GNB2L1 [68]), and axon guidance (FEZ2) [69].

Taken together, our results suggest that age, more so than diet, affected gene expression in skeletal muscle tissue of dogs in the current experiment. Similar results were noted in the brain in a previously published paper from this project [16]. Although acute changes in nutrient composition may affect a small number of genes in a transient manner, age has a prolonged effect [5], [9], [38]. This experiment has identified age- and diet-induced gene expression changes in canine skeletal muscle, providing a useful foundation for future experiments designed to study muscle aging. Applications could include nutraceutical development for the protection from age-related skeletal muscle function and strength decline. To more precisely assess the effects of such compounds, long-term studies analyzing skeletal muscle biopsy samples over the life span of the animal may be useful. As our knowledge of gene function and gene-gene interaction expands, nutritional supplements designed to influence specific targets may be designed.

## Materials and Methods

### Animals and diets

The University of Illinois Animal Care and Use committee approved all animal care procedures prior to initiation of this experiment. Twelve female beagle dogs were used in this experiment. Six dogs were geriatric (average age: 11.1 yr at the start of the experiment; Kennelwood Inc., Champaign, IL) and six dogs were of weanling age (8 wk at the start of the experiment; Marshall Farms USA, Inc., North Rose, NY). Dogs were housed in temperature controlled rooms (22.2°C) in individual kennels (1.1 m×0.9 m), with a 12-h light∶12-h dark cycle at the Edward R. Madigan Laboratory on the University of Illinois campus. At time of tissue collection, the mean age of the geriatric dogs was 12 y, and that of the young adults was 14 mo.

Three dogs of each age group were assigned to one of two dietary treatments. Complete ingredient and chemical composition is presented in Table 5. Briefly, one diet was formulated with animal-based protein sources (APB) and the second diet was formulated with plant-based protein sources (PPB). Both diets were formulated to meet or exceed nutrient requirements for growing dogs according to the Association of American Feed Control Officials [70]. The PPB diet was designed to have lower nutrient digestibility compared to the APB diet, and had a lower caloric density. Diets were produced in dry, kibble form by Wenger Manufacturing Company (Sabetha, KS) and fed for 12 mo. Young adult dogs were fed ad libitum to support growth and the geriatric dogs were fed to maintain initial BW throughout the experiment.

**Table 5:** Ingredient and chemical composition of diets fed to young adult and geriatric dogs for 12 months.

Ingredient	APB^1^	PPB^2^
	%, *as-is*	
Corn	-	45.00
Brewers rice	44.23	-
Poultry byproduct meal	32.91	-
Soybean meal	-	19.96
Poultry fat	14.99	3.97
Wheat middlings	-	13.20
Meat and bone meal	-	10.00
Beet pulp	4.00	4.00
Dehydrated egg	2.20	2.20
Sodium chloride	0.65	0.65
Potassium chloride	0.65	0.65
Choline chloride	0.13	0.13
Vitamin premix^3^	0.12	0.12
Mineral premix^3^	0.12	0.12
Analyzed composition		
Dry matter, %	93.8	94.3
	*% DM-basis*	
Organic matter	92.8	92.3
Ash	7.2	7.7
Crude protein	28.0	25.5
Acid hydrolyzed fat	22.6	11.2
Total dietary fiber	4.8	15.2
Gross energy, *kJ/g*	22.5	19.8

1 Provided per kg of APB diet: choline, 2654 mg; retinyl acetate, 15.2 KIU; cholecalciferol, 0.9 KIU; 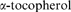, 62.5 IU; menadione sodium bisulfite complex (source of vitamin K), 0.6 mg; thiamin, 13.1 mg; riboflavin, 14.0 mg; pantothenic acid, 25.3 mg; niacin, 70.0 mg; pyridoxine, 13.56 mg; biotin, 0.11 mg; folic acid, 949 µg; vitamin B-12, 129 µg; manganese (as MnSO_4_), 19.6 mg; iron (as FeSO_4_), 253.9 mg; copper (as CuSO_4_), 17.8 mg; cobalt (as CoSO_4_), 2.4 mg; zinc (as ZnSO_4_), 166.9 mg; iodine (as KI), 6.3 mg; and selenium (as Na_2_SeO_3_), 0.32 mg.

2 Provided per kg of PPB diet: choline, 2457 mg; retinyl acetate, 16.3 KIU; cholecalciferol, 0.9 KIU; α-tocopherol, 74.1 IU; menadione sodium bisulfite complex (source of vitamin K), 1.2 mg; thiamin, 14.4 mg; riboflavin, 11.5 mg; pantothenic acid, 23.9 mg; niacin, 79.3 mg; pyridoxine, 15.8 mg; biotin, 0.24 mg; folic acid, 1024 µg; vitamin B-12, 33.3 µg; manganese (as MnSO_4_), 24.0 mg; iron (as FeSO_4_), 214.6 mg; copper (as CuSO_4_), 23.1 mg; cobalt (as CoSO_4_), 2.4 mg; zinc (as ZnSO_4_), 144.3 mg; iodine (as KI), 24.0 mg; selenium (as Na_2_SeO_3_), 0.27 mg.

3 Trouw Nutrition USA, LLC, Highland, IL.

### Tissue collection and RNA extraction

After 12 mo on experiment, dogs were euthanized after a 12-h fast by administering a lethal dose of sodium pentobarbital (130 mg/kg BW; Euthasol, Virbac Corp., Forth Worth, TX). After death was confirmed by lack of a pulse, corneal reflex, and heartbeat, muscle tissue samples were immediately taken from the biceps femoris and flash-frozen in liquid nitrogen. Tissue samples were stored at −80°C until further processing.

Total cellular RNA was extracted from tissue samples using the Trizol method according to manufacturer's instructions (Invitrogen, Carlsbad, CA). RNA was quantified on an ND-1000 spectrometer (Nanodrop Technologies, Wilmington, DE) and RNA quality verified on 1.2% agarose gels. All individual tissue RNA samples were analyzed individually to assess inter-animal variation.

### Microarray procedure

All RNA samples were analyzed by hybridization to the Affymetrix GeneChip® Canine Genome Arrays (Affymetrix, Santa Clara, CA). Hybridization reactions were performed using Affymetrix GeneChip Expression 3′-Amplification Reagents (One-Cycle Target Labeling and Control Reagents package) according to manufacturer instructions and as described by Swanson et al. [16].

After hybridization, the microarray chips were washed and stained using a streptavidin-conjugated phycoerythrin dye (Invitrogen) enhanced with biotinylated goat anti-streptavidin antibody (Vector Laboratories, Burlingame, CA) using an Affymetrix GeneChip Fluidics Station 450 and Genechip Operating Software. Images were scanned using an Affymetrix GeneChip scanner 3000.

### Microarray data analysis

We used Affymetrix's Canine Genome array (23,836 total probes) to interrogate approximately 21,700 transcripts for *C. familiaris* obtained from GenBank (August 2003), dbEST (October 2003), and cDNA libraries from 11 tissues, including skeletal muscle, licensed from LION bioscience AG. Quality control measures included Affymetrix's recommended measures, as well as assessments using the affy [71], affyPLM [72], and made4 [73] packages from the Bioconductor project [74]. All of the arrays passed quality control.

Each probe set contained 11 perfect match (PM) and 11 mismatch (MM) probes. The raw PM and MM probe-level data were pre-processed into one number per probe set using the GCRMA algorithm in Bioconductor's affy [71] and GCRMA [75] packages. GCRMA does a GC-content-based background correction, performs quantile normalization and then summarizes the PM values into one number using median polish. Because the Canine Genome array contains transcripts from many tissues, we used Affymetrix's call detection algorithm [76] to assess which probe sets were reliably detected above background on each array. Probe sets were discarded from further analysis if they were not called present on at least one array or marginal on two arrays. Of the 23,836 total probe sets, 13,405 passed this filter and were assessed for differential expression due to age and diet (described below). Heat maps were generated using the Heatplus [77] package from Bioconductor [74]. Meta-Core (GeneGo, Inc., St. Joseph, MI) was used to build gene networks and interpret microarray data. Functional attribution was made according to the database SOURCE (http://source.stanford.edu) [78].

### qRT-PCR analysis

Quanitative reverse transcriptase-polymerase chain reaction (qRT-PCR) was used to validate a subset of genes expressed differentially based on microarray results. Gene-specific primer-probe pairs were designed for each gene using Primer Express 2.0 software (PerkinElmer, Boston, MA). cDNA was prepared from tissue RNA samples using the High Capacity cDNA Archive Kit (Applied Biosystems, Foster City, CA). cDNA samples then were evaluated using real-time two-step qRT-PCR using an Applied Biosystems Taqman Gene Expression Assay containing a FAM dye-labeled Taqman MGB probe on the Applied Biosystems 7900HT Real-Time PCR System. Gene validation was done in triplicate using a control sample to assemble a standard curve. Eukaryotic 18S rRNA was amplified as a control in parallel with the gene of interest. After amplification, data were normalized to 18S rRNA and expressed as a ratio to the 18S rRNA signal.

### Statistical analysis

Differential expression of the microarray data was evaluated using the limma package [79]. A linear model for the four age×diet groups was fit for each probe set. Differences between groups were then extracted from the model as contrasts. An empirical Bayes “shrinkage” method was employed on the standard errors to improve power for small sample sizes [79]. Last, multiple test correction of P-values was done using the false discovery rate (FDR) method [80]. qRT-PCR data were analyzed using the General Linear Models procedure of SAS (SAS Institute., Cary, NC). Differences were considered significant when P<0.05.

## Supporting Information

Table S1 Complete list of skeletal muscle gene expression in old vs. young adult dogs fed diets formulated with mainly animal-based protein (APB) sources or mainly plant-based protein (PPB) sources (annotated genes only).
(0.28 MB DOC)
