# Supplementary material for: Age and Diet Affect Gene Expression Profile in Canine Skeletal Muscle
Source: PLoS One. 2009 Feb 16;4(2):e4481. doi: 10.1371/journal.pone.0004481 (PMC2637985; doi:10.1371/journal.pone.0004481)
Supplement: Table S1 — Complete list of skeletal muscle gene expression in old vs. young adult dogs fed diets formulated with mainly animal-based protein (APB) sources or mainly plant-based protein (PPB) sources (annotated genes only). (0.28 MB DOC) [file pone.0004481.s001.doc]

| **Table S1. Complete list of skeletal muscle gene expression in old vs. young adult dogs fed diets formulated with mainly animal-based protein (APB) sources or mainly plant-based protein (PPB) sources (annotated genes only).** | | | | |
| --- | --- | --- | --- | --- |
| **Functional classification** | **Gene name** | **Gene symbol** | **Fold change** | |
|  |  |  | **APB** | **PPB** |
| **Cell cycle and apoptosis** |  |  |  |  |
| Actin cytoskeleton organization and development | Phosphodiesterase 4d interacting protein (myomegalin) | PDE4DIP | 1.30 | 1.90* |
| Apoptosis | Diablo homolog (drosophila) | DIABLO | -1.25 | -2.18* |
| Apoptosis | Inhibitor of growth family, member 4 | ING4 | -1.23 | -1.59* |
| Apoptosis | B-cell receptor-associated protein 31 | BCAP31 | -1.47 | -1.80* |
| Apoptosis | Autophagy related 12 homolog (s. Cerevisiae) | ATG12 | -1.08 | -1.42* |
| Apoptosis | Mitochondrial ribosomal protein S30 | MRPS30 | 1.63* | 1.56 |
| Apoptosis | Zinc finger, DHHC-type containing 16 | ZDHHC16 | -1.45* | -1.31 |
| Apoptosis | TIMP metallopeptidase inhibitor 3 | TIMP3 | 1.36 | 1.90* |
| Carcinogenesis | Melanoma antigen family d, 2 | MAGED2 | -1.53 | -1.80* |
| Cell adhesion | Heparan sulfate proteoglycan 2 | HSPG2 | -1.36 | -1.95* |
| Cell adhesion | Digeorge syndrome critical region protein 6 | DGCR6 | -1.63 | -1.84* |
| Cell adhesion | Endoglin (osler-rendu-weber syndrome 1) | ENG | -2.85* | -3.61* |
| Cell aging | LIM and senescent cell antigen-like domains 1 | LIMS1 | 1.31 | 3.13* |
| Cell cycle | Transforming growth factor, beta 1 | TGFB1 | -1.54 | -2.12* |
| Cell cycle | Y-box protein ZONAB-A | ZONAB | 1.39* | 1.24 |
| Cell cycle | Septin 11 | SEPT11 | 2.52* | 3.79* |
| Cell cycle | Stromal interaction molecule 1 | STIM1 | -1.60* | -1.95* |
| Cell development | Dystrophin (muscular dystrophy, Duchenne and Becker types) | DMD | 2.11* | 2.60 |
| Cell growth | Myotrophin | MTPN | 1.51 | 1.88* |
| Cell motility | Dynein, axonemal, heavy polypeptide 9 isoform 2 | DNAH9 | -1.18 | -1.62* |
| Cell proliferation | Arginine/serine-rich coiled-coil 2 | RSRC2 | 1.83 | 4.62* |
| Cell proliferation | RAS homolog gene family, member g (rho g) | RHOG | -1.04 | -1.65* |
| Cell proliferation | Serpin peptidase inhibitor, clade F (alpha-2 antiplasmin, pigment epithelium derived factor), member 1 | SERPINF1 | -2.28* | -1.84 |
| Cell signaling | Cerebral cavernous malformation 2 | CCM2 | -1.41 | -1.45* |
| Cell survival | Acidic (leucine-rich) nuclear phosphoprotein 32 family, member b | ANP32B | 1.43 | 1.74* |
| Cell survival | Hcls1 associated protein x-1 | HAX1 | -1.56* | -1.80* |
| Cellular copper homeostasis | Metallothionein 2A | MT2A | 18.51* | 3.40 |
| Chromsome organization | Structural maintenance of chromosomes 5 | SMC5 | 1.18 | 3.17* |
| Cytokinesis | Myosin-10 (cellular myosin heavy chain, type b) | MYH10 | 3.19 | 6.22* |
| Cytoskeletal organization | Actin binding lim protein 1 | ABLIM1 | 2.23 | 3.17* |
| Cytoskeleton organization | hook homolog 2 (Drosophila) | HOOK2 | -2.02* | -1.21 |
| Differentiation | Endothelial pas domain protein 1 | EPAS1 | -1.69* | -2.10* |
| Endo/exocytosis | Rab9 effector p40 | RABEPK | -1.18 | -2.16* |
| Er-golgi transport | Trafficking protein particle complex 1 | TRAPPC1 | -1.44 | -2.08* |
| Extracellular matrix organization | Collagen, type iv, alpha 2 | COL4A2 | -1.31 | -1.62* |
| I-kappab kinase regulation | Nedd4 family interacting protein 1 | NDFIP1 | 1.29 | 2.10* |
| Intracellular signalling cascade | Ras p21 protein activator 4 | RASA4 | -1.25 | -1.61* |
| Intracellular signalling cascade | Solute carrier family 9 (sodium/hydrogen exchanger), member 3 regulator 2 | SLC9A3R2 | -2.06 | -3.73* |
| Meiotic recombination | Kelch domain containing 3 | KLHDC3 | -1.57* | -1.77* |
| Mitogenesis | Fibp fibroblast growth factor (acidic) intracellular binding protein | FIBP | -1.14 | -1.57* |
| Mitosis | Dynactin 3 (p22) | DCTN3 | -1.18 | -1.52* |
| Mitosis | Protein (peptidylprolyl cis/trans isomerase) nima-interacting 1 | PIN1 | -1.22 | -1.95* |
| Mitotic chromosome condensation | Cytoplasmic linker associated protein 2 | CLASP2 | 1.12 | 2.21* |
| Proliferation | Calpain, small subunit 1 | CAPNS1 | -1.36* | -1.41* |
| Receptor activity | Anthrax toxin receptor 2 | ANTXR2 | 1.18 | 1.62* |
| Signal transduction | Nucleolar complex associated 3 homolog (s. Cerevisiae) | NOC3L | -1.94 | -2.41* |
| Signal transduction | A kinase (prka) anchor protein 8 | AKAP8 | 1.03 | 1.33* |
| Signal transduction | Guanine nucleotide binding protein (g protein), beta polypeptide 2-like 1 | GNB2L1 | -1.19 | -1.41* |
| Signal transduction | Reticulon 2 | RTN2 | -1.35 | -1.44* |
| Signal transduction, cell surface receptor-linked | Leukemia inhibitory factor receptor alpha | LIFR | 1.01 | 2.33* |
| **Cellular organization and development** | |  |  |  |
| Development | MYBPC1 myosin binding protein C, slow type | MYBPC1 | 1.82 | 4.14* |
| Development | Nebulin | NEB | 1.86 | 3.16* |
| Development | Titin | TTN | 1.96 | 8.95* |
| Development | Myocyte enhancer factor 2C | MEF2C | 1.38 | 4.90* |
| Development | Integrin beta 1 binding protein (melusin) 2 | ITGB1BP2 | -2.02 | -2.28* |
| Development | Actin, gamma 2, smooth muscle, enteric | ACTG2 | -2.72 | -6.73* |
| Structure | DMN desmuslin | DMN | 2.13 | 8.41* |
| **Immune and stress response** |  |  |  |  |
| Anti tumor | Dermatan sulfate epimerase | DSE | 4.81* | 4.49* |
| Humoral immune response | Adenosine deaminase | ADA | -2.21* | -2.58* |
| Immune response | Gamma-interferon inducible lysosomal thiol reductase precursor | IFI30/GILT | -4.70* | -5.30* |
| Immune system | HLA-B associated transcript 5 | BAT5 | -1.45 | -1.69* |
| Immune system/housekeeping | Dom-3 homolog Z (C. elegans) | DOM3Z | -1.38 | -2.33* |
| Oxidative stress response | Peroxiredoxin 5 | PRDX5 | -1.90* | -1.90* |
| Response to virus | Hepatitis B virus x interacting protein | HBXIP | -1.21 | -1.36* |
| Response to virus | Barrier to autointegration factor 1 | BANF1 | 1.88 | 5.22* |
| Stress response | ERBB receptor feedback inhibitor 1 | ERRFI1 | 1.03 | 2.06* |
| **Metabolism** |  |  |  |  |
| Aerobic respiration | Inner membrane protein, mitochondrial precursor | OXA1L | -1.29 | -2.13* |
| Amine/lipid/steroid metabolism | Sulfotransferase family, cytosolic, 1A, phenol-preferring, member 1 | SULT1A1 | -1.31 | -2.19* |
| Amino acid metabolism | Cysteine conjugate-beta lyase; cytoplasmic (glutamine transaminase K, kyneurenine aminotransferase) | CCBL1 | -2.48* | -2.08* |
| ATP binding | RIOK1 RIO kinase 1 (yeast) | RIOK1 | 1.36 | 2.77* |
| ATP binding | Dynein light intermediate chain 2, cytosolic (LIC53/55) (LIC-2) | DYNC1LI2 | 1.42 | 3.59* |
| Calcium transport | ATPase, Ca++ transporting, plasma membrane 1 | ATP2B1 | -1.02 | 1.53* |
| Carbohydrate metabolism | ER degradation enhancer, mannosidase alpha-like 2 | EDEM2 | -1.36* | -1.66* |
| Catalytic activity/metabolism | Isochorismatase domain containing 2 | ISOC2 | -1.85 | -2.12* |
| Diabetes/obesity | Fat mass and obesity associated | FTO | 1.02 | 1.34* |
| Electron transport | NADH dehydrogenase (ubiquinone) 1 beta subcomplex, 9, 22kDa | DNDUFB9 | -1.29 | -1.37* |
| Electron transport | Isovaleryl Coenzyme A dehydrogenase | IVD | -1.50 | -1.69* |
| Electron transport | NADH dehydrogenase (ubiquinone) Fe-S protein 6, 13kDa (NADH-coenzyme Q reductase) | NDUFS6 | -1.22 | -1.42* |
| Electron transport chain | NADH dehydrogenase 1 beta subcomplex, 11, 17.3kda | NDUFB11 | -1.61* | -1.71* |
| Electron transport chain | Ring finger 144b | RNF144B | -2.79* | -2.56* |
| Electron transport chain | Glutaryl-Coenzyme A dehydrogenase | GCDH | -2.01* | -1.50 |
| Energy metabolism | NADH dehydrogenase (ubiquinone) 1 alpha subcomplex, 10, 42kDa | NDUFA10 | -1.66* | -1.46 |
| Fatty acid metabolism | Protein kinase, AMP-activated, gamma 1 non-catalytic subunit | PRKAG1 | -1.41 | -2.10* |
| Glutathione conjugation | Glutathione S-transferase P (GST 7-7) (Chain 7) (GST class-pi) | GSTP1 | -1.22 | -1.77* |
| Glycolysis | Glyceraldehyde-3-phosphate dehydrogenase | GAPDH | -1.13 | -2.13* |
| Glycolysis | Aldolase A, fructose-bisphosphate | ALDOA | 1.53 | 2.54* |
| Heme biosynthesis | Heme a:farnesyltransferase | COX10 | -1.57* | -1.54* |
| Hydrolase activity/metabolism | Cat eye syndrome chromosome region, candidate 5 isoform 2 precursor | CECR5 | -1.31 | -1.77* |
| Intracellular protein transport | ADP-ribosylation factor 3 | ARF3 | 1.01 | -1.18* |
| Intracellular protein transport | ADP-ribosylation factor 5 | ARF5 | -1.31 | -1.68* |
| Leucine catabolism | Methylcrotonoyl-Coenzyme A carboxylase 2 (beta) | MCCC2 | -2.22* | -1.14 |
| Lipid metabolism | Hydroxysteroid (17-beta) dehydrogenase 10 | HSD17B10 | -1.76* | -1.83* |
| Lipid metabolism | Microsomal glutathione S-transferase 3 | MGST3 | -1.60 | -1.87* |
| Nucleoside biosynthesis | Non-metastatic cells 6, protein expressed in (nucleoside-diphosphate kinase) | NME6 | -1.41 | -3.05* |
| Oxidation of organic compounds | BCS1-like (yeast) | BCS1L | -1.38 | -1.82* |
| Protein AA dephosphorylation | Dual specificity phosphatase 16 | DUSP16 | -1.37 | -1.64* |
| Protein AA dephosphorylation | Protein phosphatase 2, regulatory subunit B'', alpha | PPP2R3A | 1.30 | 3.67* |
| Protein AA dephosphorylation | Palladin, cytoskeletal associated protein | PALLD | 1.08 | 1.30* |
| Protein AA phosphorylation | Janus kinase 1 (a protein tyrosine kinase) | JAK1 | 1.66 | 3.64* |
| Protein AA phosphorylation | Casein kinase 2, alpha 1 polypeptide | CSNK2A1 | 1.11 | 1.51* |
| Protein AA phosphorylation | Activin A receptor, type IIA | ACVR2A | 1.05 | 1.33* |
| Protein AA phosphorylation | Branched chain ketoacid dehydrogenase kinase | BCKDK | -1.47 | -2.00* |
| Protein AA phosphorylation | Mixed lineage kinase-related kinase MRK-beta isoform 2 | MLKL | 2.00 | 2.66* |
| Protein assembly | WD repeat domain 45 | WDR45 | -1.25 | -1.56* |
| Protein binding | Kelch repeat and BTB (POZ) domain containing 4 | KBTBD4 | -1.22 | -1.78* |
| Protein binding | 78 kDa glucose-regulated protein precursor (GRP 78) (Endoplasmic reticulum lumenal Ca(2+) binding protein grp78) | HSPA5 | 1.69 | 2.69* |
| Protein biosynthesis | Deoxyhypusine synthase | DHPS | -1.45 | -2.03* |
| Protein biosynthesis | Mitochondrial ribosomal protein L14 | MRPL14 | -1.37 | -1.65* |
| Protein biosynthesis | Mitochondrial ribosomal protein S18A | MRPS18A | -1.34 | -1.72* |
| Protein biosynthesis | Ribosomal protein S16 | RPS16 | -1.15 | -2.00* |
| Protein biosynthesis | Ribosomal protein L19 | RPL19 | -1.03 | -1.67* |
| Protein biosynthesis | Mitochondrial ribosomal protein S12 | MRPS12 | -1.42 | -1.75* |
| Protein biosynthesis | 40S ribosomal protein S10 | ? | -1.22 | -1.39* |
| Protein catabolism | Spastic paraplegia 7 (pure and complicated autosomal recessive) | SPG7 | -1.42 | -2.00* |
| Protein catabolism | Ubiquitin-conjugating enzyme E2D 2 | UBE2D2 | -1.12 | -1.91* |
| Protein catabolism | Ubiquitin specific peptidase 16 | USP16 | 1.22 | 3.23* |
| Protein folding | HECT domain containing 1 | HECTD1 | 2.84 | 7.46* |
| Protein folding | Heat shock protein 1, beta | HSPD1 | 1.68 | 2.50* |
| Protein folding | Ubiquitously-expressed transcript | UXT | -1.11 | -1.81* |
| Protein folding | Heat shock protein HSP 90-alpha (HSP 86) | HSP90AA1 | 1.50 | 2.69* |
| Protein localization | CutA divalent cation tolerance homolog (E. coli) | CUTA | -1.53 | -1.91* |
| Protein metabolism | Coenzyme Q7 homolog, ubiquinone (yeast) | COQ7 | -1.68 | -1.72* |
| Protein modification | Ubiquitin-like 4A | UBL4A | -2.29* | -1.52 |
| Protein phosphatase inhibition | Protein phosphatase 1, regulatory (inhibitor) subunit 11 | PPP1R11 | -1.36 | -1.70* |
| Protein processing | Presenilin enhancer 2 homolog (C. elegans) | PSENEN | -1.37 | -1.93* |
| Protein transport | Importin 9 | IPO9 | 1.44 | 2.70* |
| Protein transport | Translocase of outer mitochondrial membrane 22 homolog (yeast) | TOMM22 | -1.31 | -1.50 |
| Protein ubiquitination | Membrane-associated ring finger (C3HC4) 2 isoform 1 | MARCH2 | -1.38 | -1.62* |
| Protein ubiquitination | Ring finger protein 181 | RNF181 | -1.10 | -1.60* |
| Protein ubiquitination | F-box and WD repeat domain containing 11 | FBXW11 | 1.50 | 2.50* |
| Proteolysis | Peptidase (mitochondrial processing) alpha | PMPCA | -1.38 | -1.54* |
| Serine biosynthesis | Glyoxylate reductase/hydroxypyruvate reductase | GRHPR | -1.37 | -1.73* |
| TCA cycle | Isocitrate dehydrogenase 3 (NAD+) beta | IDH3B | -2.19* | -2.23* |
| TCA cycle | Succinate dehydrogenase complex, subunit C, integral membrane protein, 15kDa | SDHC | -1.43* | -1.34 |
| Ubiquitin cycle | Ubiquitin-conjugating enzyme E2E 1 (UBC4/5 homolog, yeast) | UBE2E1 | 1.43* | 1.20 |
| **Transcription – translation** |  |  |  |  |
| DNA binding | CGG triplet repeat binding protein 1 | CGGBP1 | -1.67 | -1.65* |
| DNA damage response | Sestrin 1 | SESN1 | 2.02* | 1.43 |
| DNA replication | Ubiquitin-like modifier activating enzyme 1 | UBE1 | -1.17 | -1.60* |
| DNA structure | Polymerase (DNA-directed), epsilon 4 (p12 subunit) | POLE4 | -1.20* | -1.32* |
| Mrna binding | Unc-50 homolog (C. elegans) | UNC50 | -1.14 | -1.60* |
| mRNA processing | Small nuclear ribonucleoprotein polypeptide N | SNRPN | -1.54* | -1.73* |
| Mrna processing | Heterogeneous nuclear ribonucleoprotein A1 | HNRNPA1 | 1.28 | 1.36* |
| Mrna splicing | PRP40 pre-mRNA processing factor 40 homolog A (S. cerevisiae) | PRPF40A | 1.44 | 2.47* |
| Nucleic acid binding | DEAH (Asp-Glu-Ala-His) box polypeptide 29 | DHX29 | 1.35 | 3.24* |
| Nucleic acid binding | G patch domain containing 1 | GPATCH1 | 1.00 | -1.19* |
| Nucleic acid binding | Zinc finger CCCH-type containing 11A | ZC3H11A | 1.12 | 5.09* |
| Nucleic acid binding | Quaking homolog, KH domain RNA binding (mouse) | QKI | 1.26 | 2.08* |
| Processing | Heterogeneous nuclear ribonucleoprotein H1 (H) | HNRPH1 | -1.09 | 2.97* |
| Processing | Nucleolar protein family A, member 3 (H/ACA small nucleolar RNPs) | NOLA3 | -1.28 | -1.55* |
| Ribosome assembly | Nucleolar protein family a, member 2 | NOLA2 | -1.36* | -1.89* |
| RNA polymerase II promoter transcription regulator | Heat shock factor binding protein 1 | HSBP1 | -1.15 | -1.50* |
| Splicing | Synaptotagmin binding, cytoplasmic RNA interacting protein | SYNCRIP | 1.80 | 4.00* |
| Splicing | Splicing factor, arginine/serine-rich 5 | SFRS5 | 2.13 | 2.24* |
| Transcription regulation | RNA polymerase II CTD phosphatase homolog (S. cerevisiae) | SSU72 | -1.25 | -1.59* |
| Transcription regulation | Mediator complex subunit 31 | MED31 | -1.14 | -1.94* |
| Transcription regulation | Retinoblastoma-like 2 (p130) | RBL2 | 1.61 | 3.45* |
| Transcription regulation | Zinc finger protein 207 | ZNF207 | -1.17 | -1.63* |
| Transcription regulation | Bromodomain adjacent to zinc finger domain, 2B | BAZ2B | 1.17 | 2.44* |
| Transcription regulation | YY1 transcription factor | YY1 | 1.22 | 2.22* |
| Transcription regulation | MBT domain containing 1 | MBTD1 | 1.10 | 1.73* |
| Transcription regulation | PWP1 homolog (S. cerevisiae) | PWP1 | 2.04 | 3.10* |
| Transcription regulation | Jumonji, AT rich interactive domain 1A | JARID1A | 1.00 | 1.31* |
| Transcription regulation | Silent mating type information regulation 2 homolog 3 (S. cerevisiae) | SIRT3 | -1.67 | -2.18* |
| Transcription regulation | Retinoic acid receptor, beta | RARB | 1.03 | 2.79* |
| Transcription regulation | MYC associated factor X | MAX | -1.13 | -1.67* |
| Transcription regulation | Alpha thalassemia/mental retardation syndrome X-linked (RAD54 homolog, S. cerevisiae) | ATRX | 1.72 | 3.54* |
| Transcription regulation | BolA homolog 1 (E. coli) | BOLA1 | -1.36 | -1.91* |
| Transcription regulation | cAMP responsive element modulator | CREM | -2.95* | -2.47 |
| Transcription regulation | Zinc finger protein 32 | ZNF32 | -1.63* | -1.75* |
| Translation | DEAD (Asp-Glu-Ala-Asp) box polypeptide 42 | DDX42 | 1.08 | 2.06* |
| **Signaling mechanisms** |  |  |  |  |
| Cellular trafficking | Methylmalonic aciduria cblc type, with homocystinuria | MMACHC | -2.21* | -2.15* |
| Intracellular signaling cascade | A kinase (prka) anchor protein 13 | AKAP13 | 1.96* | 1.86* |
| Neurotransmission | Rab3a, member ras oncogene family | RAB3A | -2.27* | -2.22* |
| Neurotransmission | RIMS binding protein 2 | RIMBP2 | 1.25* | -1.01 |
| **Transport** |  |  |  |  |
| Nuclear protein import | TPR translocated promoter region (to activated MET oncogene) | TPR | 1.30 | 3.53* |
| Peptide transport | Magmas-like protein | TIM16 | -1.48* | -1.97 |
| Protein transport | Sec61 alpha 2 subunit (s. Cerevisiae) | SEC61A2 | -1.54* | -1.45* |
| Taurine transport | Solute carrier family 6 (neurotransmitter transporter), member 6 | SLC6A6 | 1.87* | 2.40* |
| Transport | Solute carrier family 25, member 33 | SLC25A33 | -3.14* | -3.42* |
| UDP-galactose transport | Solute carrier family 35, member B1 | SLC35B1 | -1.28 | -2.04* |
| Unknown | Solute carrier family 25, member A26 | SLC25A26 | -1.21 | -2.13* |
| Unknown | Mitochondrial carrier homolog 2 (C. elegans) | MTCH2 | -1.25 | -1.40* |
| Unknown | ATP-binding cassette, sub-family A (ABC1), member 5 | ABCA5 | 1.33 | 2.45* |
| **Other** |  |  |  |  |
| Catalytic activity | Sel-1 suppressor of lin-12-like (C. elegans) | SEL1L | 1.94 | 3.01* |
| Catalytic activity | Isoamyl acetate-hydrolyzing esterase 1 homolog (S. cerevisiae) | IAH1 | -1.43 | -1.90* |
| GPI-anchor biosynthesis | Phosphatidylinositol glycan anchor biosynthesis, class C | PIGC | -1.10 | -1.57* |
| Methionine catabolism | Sulfite oxidase | SUOX | -1.39 | -2.17* |
| Neural development | Dullard homolog (Xenopus laevis) | DULLARD | -1.50 | -1.83* |
| Organogenesis | Glycophorin C (Gerbich blood group) | GYPC | -1.56* | -1.60 |
| Part of ribosome structure | Mitochondrial ribosomal protein L43 | MRPL43 | -1.46 | -1.99* |
| Vision | Hippocalcin-like 1 | HPCAL1 | -1.66* | -1.30 |
